# Rethinking cost-effectiveness in the era of zero healthcare spending growth

**DOI:** 10.1186/s12939-016-0326-8

**Published:** 2016-02-24

**Authors:** Ronen Arbel, Dan Greenberg

**Affiliations:** Department of Health Systems Management, Faculty of Health Sciences and The Guilford-Glazer Faculty of Business and Management, Ben-Gurion University of the Negev, Beer-Sheva, Israel; Department of Technology Marketing, Sapir Academic College, M. P. Hof Ashkelon 7919, Sderot, Israel

**Keywords:** Health Equity, Budget Impact, Cost-Effectiveness, Decision Making, Economic Outcome

## Abstract

**Background:**

The global economic crisis imposes severe restrictions on healthcare budgets, limiting the coverage of new interventions, even when they are cost-effective. Our objective was to develop a tool that can assist decision-makers in comparing the impact of medical intervention alternatives on the entire target population, under a pre-specified budget constraint.

**Methods:**

We illustrated the tool by using a target population of 1,000 patients, and a budget constraint of $1,000,000. We compared two intervention alternatives: the current practice that costs $1,000 and adds 0.5 quality-adjusted-life-years (QALYs) per patient and a new technology that costs 100 % more, and provides 20 % more QALYs per patient. We also developed a formula for defining the maximum premium price for a higher-cost/higher-effectiveness intervention that can justify its adoption under a constrained budget.

**Results:**

Using the new therapy will add 300 QALYs, compared to 500 QALYS when using the lower-cost, lower-effective intervention, despite a favorable incremental cost-effectiveness ratio (ICER) of $10,000. The maximum price for the higher-efficacy therapy that will preserve the target population outcomes is 20 % higher than the lower-cost therapy.

**Conclusions:**

Although an intervention associated with higher costs and higher efficacy may have an acceptable ICER, it could provide inferior outcomes in the target population under budget constraints, depending on the relative effectiveness and costs of the interventions. The cost premium that can be justified for a higher-efficacy intervention is directly correlated to its effectiveness premium. Using the proposed tool may assist decision-makers in improving overall healthcare outcomes, especially in times of economic downturn.

## Background

### Promoting health equity can improve the population’s overall health outcomes

**Fig. 1:**
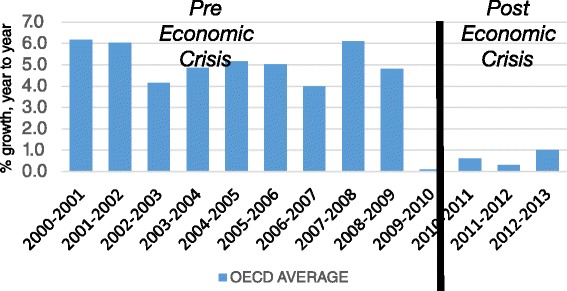
Annual Growth in public expenditures, Average OECD

The use of innovative medical technologies has significantly improved population’s health outcomes, but this improvement came with escalating healthcare costs [1]. The recent global economic crisis imposed severe restrictions on public budgets allocated to healthcare and health spending growth has leveled off in many countries in the last few years, as shown in Fig. 1 [2]. Thus, there is a need to continue the improvement of health outcomes while containing health budgets. Current healthcare policies, however, are very far from maximizing the health outcomes of the population within the given budgets. One option for achieving this goal is to reallocate resources to interventions that provide the most “value for money”. In this regard, Chambers et al. [3] have recently suggested that more effective allocation of budgets may yield an additional 1.8 million quality-adjusted life-years (QALYs) in the US Medicare population, while maintaining the same spending. Another policy may be providing the population with slightly less effective medical interventions but at significantly lower cost, thus enabling effective treatment for a wider population. A recent study has presented the advantages of using this approach, by analyzing the utilization patterns of biologic drugs to avoid blindness due to age-related macular degeneration or clinically significant diabetic macular edema [4]. The authors demonstrated potential savings of $29B over the course of 10 years by using a forty times less expensive drug (bevacizumab), which has minor differences in adverse events.

One could argue that it is mandatory for physicians to provide the best available medicine to their patients, or at least inform them that such a medicine is available. However, the counter claim is that from a broader societal perspective, it is more important to achieve equity in the supply of medical innovations. These claims have previously been observed and discussed in the Prospective Urban Rural Epidemiological (PURE) study [5]. The study recruited individuals in countries at various stages of economic development, to assess the rates of use of proven and effective secondary cardiovascular prevention drugs. The authors concluded that because the use of secondary prevention medications is low worldwide, especially in low-income countries and rural areas, systematic approaches are needed to improve the long-term use of basic, inexpensive and effective drugs. Although lower quality, lower cost products are very common in many markets (e.g., consumer electronics, car safety measures, furniture), barriers remain for using such products in health care. Indeed, only very few cost-effectiveness analyses demonstrated cost-savings while providing (acceptable) inferior outcomes [5], and this approach is not implemented in most developed countries.

In this study, we first review the limitations of current health technology assessment (HTA) models in providing decision-makers with tools that may assist them in maximizing population’s health under restricted budgets. We then develop an analytic framework for comparing and assessing the impact of alternative interventions under pre-specified budget constraints; one providing an effective and expensive intervention to a limited patient population and the other providing slightly less effective but substantially cheaper intervention to a wider target population. We argue that a widespread use of less effective and lower cost treatment alternatives may provide better health outcomes to the society. Our proposed model is probably one of the first to combine both the cost-effectiveness and the budget impact of an alternative.

### Limitations of cost-effectiveness analysis

Economic analyses and decisions on adoption of innovative technologies have an important impact on society's budgets and well-being. Health technology assessment (HTA) assesses the short and long-term consequences of using technologies in health care and provides policy-makers with information on competing policy alternatives [6]. While HTA includes various aspects related to the use of technologies in health care, most analyses focus on economic evaluations (e.g., cost-effectiveness analysis) and the resources needed to implement an intervention in clinical practice (e.g., budget impact analysis). Cost-effectiveness analysis (CEA) is an analytic tool that quantifies the added expenditure necessary to obtain a unit of health benefit, typically measured in quality-adjusted life-years (QALYs) gained. Table 1 illustrates the four quadrants of the cost-effectiveness plane. Interventions that are associated with lower costs and higher effectiveness (dominant) should be adopted, while those associated with higher costs and lower effectiveness (dominated) should be rejected. Although in many countries there is no accepted threshold value identifying alternatives deemed cost-effective, several arbitrary thresholds have been suggested [7–9], typically ranging from $50,000 to $150,000. There is no guidance on the “right” decision for interventions that are less effective and less expensive compared with an accepted alternative.

**Table 1:** Distribution of Cost-Effectiveness studies

Quadrant	Efficacy	Cost	% of CE studies
North East	Better	Higher	72 %
North West	Lower	Higher	10 %
South East	Higher	Lower	16 %
South West	Lower	Lower	2 %

For a medical innovation to be widely adopted into routine practice, a “new and improved” reputation is customarily required [10]. A review of published cost-effectiveness analyses revealed that the majority of interventions examined are associated with higher costs and better outcomes, and only approximately 2 % of CEAs present interventions that are cost-saving while providing (acceptably) inferior outcomes [5] as presented in Table 1.

The use of the current cost-effectiveness paradigm assumes that addition of QALYs usually implicates higher costs, as most cost-effectiveness analyses present better outcomes at a higher cost [5]. This implies that healthcare costs need to rise endlessly, in order to improve outcomes. The limitations of CEA are discussed in the literature for at least two decades, and have been shown to contradict welfare economic principles, especially under budget constraints [11]. Also, the use of the incremental cost-effectiveness ratio (ICER), has been criticized for increasing expenditures on healthcare technologies in an unsustainable manner [12], and it was argued that it may not the right tool to make the best use of healthcare resources [13].

Current CEA do not provide sufficient information on which interventions should be utilized to maximize population’s health outcomes, as they estimate the “value for money” for an “average” typical patient, ignoring the associated budget consequences (i.e., affordability), and also ignoring how patients might differ from one another in their benefits and/or treatment costs [14]. Since the vast majority of innovative medical interventions are associated with better outcomes, but also with increased costs when compared to the standard of care, adopting these interventions in routine clinical practice may frequently result in a need for very high additional budgets, especially when these treatments are targeted at a large patient population. This challenge has been recently demonstrated by the new therapies for Hepatitis C virus (HCV). New therapies for HCV show cure rates of 90 % and higher [15], with the cost of therapy is between $66,000 and $84,000 per patient in the US [16]. While cost-effectiveness analyses have shown favorable ICERs for these therapies in Spain [17], France [18] and prisoner population in the US [19]. However, treating 184,000,000 HCV patients worldwide, seems simply unaffordable [20].

As continued increase in health expenditures is no longer sustainable in many countries and there is a clear global need for a policy that will improve health outcomes, without increasing healthcare costs. From a societal perspective, when resources are constrained, the fundamental goal of innovation should not be restricted to an improvement on the best available option. Rather, in a setting in which some do not have access to the “best” technology, more aggregate health benefits may be achieved from a policy allowing a widespread diffusion of effective alternatives, albeit not the best ones. There is a clear need for an economic model that would account for the total health benefits for the entire intended-use (IU) population, under a constrained budget.

### Coping with limitations of cost-effectiveness analysis

Another important component of HTA is the budget impact analysis that assesses the financial consequences of the introduction of a new technology in a specific setting in the short-to-medium term [21]. Budget impact analysis is supposed to be complementary to more established types of economic evaluations, mainly cost-effectiveness analysis, by providing decision-makers with additional information on the financial consequences of covering and reimbursing new technologies. Thus, the outcomes of the budget impact analysis should reflect scenarios that consist of a set of specific assumptions and data inputs of interest to the decision-maker rather than a scientifically chosen “base” or “reference” case as is usually done in the CEA. Decision makers need to take into account both the cost –effectiveness of and the affordability of technologies, and it has been suggested to use an “affordability curve” [22] for this purpose. Another method is to try to optimize the health gains under a constrained budget. An early approach in this regard was used for treating cardiovascular disease in Sweden [23]. The authors tried to maximize the health gains, i.e. primary prevention of cardiovascular events, by calculating the optimal distribution of a fixed budget among drug treatment of hypertension, drug treatment of cholesterol and life-style intervention. A traditional league table method was used to divide the budget among these treatment options. All interventions were ranked by their ICER and a cut-off line was drawn when the budget was exhausted. In a more recent analysis, the authors used a Markov model to synthesize clinical and economic evidence and to compute population-level costs and effects of interventions in the psychiatry field [24]. The model compared a base case scenario without preventive telemedicine and alternative scenarios with preventive telemedicine. The primary outcome measure was the benefit-to-cost ratio, or return-on-investment (ROI). The researchers ran a scenario that kept the healthcare budget constant, in which the costs of offering preventive telemedicine were balanced by reducing the expenditures for curative interventions. The results demonstrated that a system with preventive telemedicine for depressive disorders offers better ROI than a healthcare system without it. A more comprehensive approach to optimize a full portfolio of healthcare programs under a fixed budget was suggested, by using a complex mathematical model [25]. The authors demonstrated the importance of taking budgetary considerations and uncertainty into account when making decisions and decision rules.

All of these models are yet to be implemented in a real world setting, perhaps as a result of their complexity. Therefore, our objective was to develop a practical framework that can assist decision-makers in comparing the impact of intervention alternatives on the entire target population, under a pre-specified budget constraint.

## Methods

### Mathematical model

We define the upper limit of the number of patients that can be assigned to a specific alternative v as the PTPv (potentially treated population). We define Cv as the direct annual cost of a specific alternative, per patient, and B as the annual budget limit. Now, we can calculate the upper limit of the number of patients that can be treated annually by alternative v, as shown in Equation 1:

1 $$ PTPv=\frac{B}{Cv} $$

If we denote the overall efficacy of each intervention as Ev, we can calculate the total events prevented by using intervention v, denoted PEPv as shown in Equation 2:

2 $$ PEPv=Ev\times PTPv $$

We can calculate PEPv as a function of Ev, Cv and B, as shown in Equation 3:

3 $$ PEPv=Ev\times PTPv=\frac{Ev*B}{Cv} $$

Since B is constant, the ratio between PEP2 and PEP1 can be calculated as shown in Equation 4:

4 $$ \frac{PEP1}{PEP2}=\frac{E1\times B}{C1}\times \frac{C2}{E2\times B}=\frac{E1\times C2}{E2\times C1}=\frac{E1}{E2}\times \frac{C2}{C1}=\frac{E1}{C1}\times \frac{C2}{E2} $$

The implication is that in order for a more expensive therapy to be more effective on the entire population, its relative effectiveness has to be higher than its relative market price.

We can also use Equation 4 to calculate the breakeven cost for a new intervention C2, by defining that PEP1 = PEP2. Using Equation 4 we can receive Equation 5:

5 $$ \frac{PEP1}{PEP2}=1=\frac{E1}{E2}\times \frac{C2}{C1}==>C1=\frac{E1}{E2}\times C2 $$

Assuming we know the ratio of superiority of E1 over E2, we can calculate the breakeven price for the expensive/effective intervention. For example, if E1 is 20 % higher than E2, than in up to 20 % higher cost it will provide better outcomes for the IUP, and in any cost higher than that it would provide inferior outcomes for the IUP. For comparison, the conventional ICER, which does not take into account budget constraints, is calculated as follows: $\frac{C2-C1}{E2-E1}$

Therefore, while the high cost/high effectiveness intervention may have an acceptable ICER, it could provide inferior outcomes under budget constraints, depending on the ratios between the efficacies and the costs of the two interventions.

### Model assumptions

We make the following assumptions in our model, in order to maintain its simplicity:

- The budget B is fixed.
- The two interventions are mutually exclusive.
- Total costs include intervention costs, and potential cost offsets.
- QALYs and costs are discounted to present values.

## Results and discussion

### Results

We demonstrate the model by using a hypothetical scenario in which the target population (IUP) includes 1,000 patients, and the budget constraint B is $1,000,000. We compared two interventions: the current technology (V = 1) that costs $1,000 per patient, and adds 0.5 QALY per patient and a new technology (V = 2) that costs 100 % more, and provides 20 % more QALYs per patient. Table 2 presents the values derived for the demo case.

**Table 2:** Results of the demo case study

Model Parameters/Intervention	Acronym	V = 1	V = 2	Delta
Budget Constraint ($US)	B	1,000,000	1,000,000	0
Cost per patient ($US)	C	1000	2,000	1000
Potentially Treated Population	PTP	1000	500	−500
Untreated Population	UTP	0	500	500
Effectiveness- as measured by added QALY per patient	E	0.50	0.60	0.10
QALYs added for the entire IUP	PEP	500	300	−200

The ICER is calculated as follows: $\frac{C2-C1}{E2-E1}.$ In this case, the ICER is $\frac{2,000-1,000}{0.6-0.5} = \$10,000.$ This ICER is well below the accepted thresholds [9] and according to the current paradigm we should adopt the new technology, as it provides good value for money. However, in our proposed model which takes into account the budget constraint, we observe a significant reduction in the overall population outcomes, as 200 QALYs will be lost if we prefer the new technology. Using equation 5, we can calculate the maximum price for the new intervention:

6 $$ C1=\frac{E1}{E2}\times C2=\frac{0.6}{0.5}\times \$1,000=\$1,200 $$

This price, as we demonstrated, is directly correlated to the effectiveness premium of the new technology, which in this case is 20 %.

### Discussion

In this study, we first reviewed the limitations of current health technology assessment models in providing decision-makers with tools that may assist them in maximizing population’s health outcomes under restricted budgets. We developed an analytic framework for comparing and assessing the impact of alternative interventions under pre-specified budget constraints. The mathematical analysis performed revealed that the added price worth paying for superior efficacy is directly related to the superior outcomes we expect to receive from the higher efficacy intervention. This insight differs significantly from traditional cost-effectiveness analysis, which may accept a much higher cost of a new therapy as being cost-effective and economically acceptable, as long as it has an acceptable ICER. Our approach resembles in this aspect the approach taken by the German Institute for Quality and Efficiency in healthcare (IQWiG), asserting that the additional costs and benefits of an intervention should be compared only to alternatives for the same indication [14]. IQWiG has used the efficiency frontier approach to represent the best outcomes that the healthcare system can achieve at current prices and efficacies of the alternative interventions. However, a major distinction exists between our proposed concept and the German approach; IQWiG is willing to consider innovative interventions only if they provide superior benefits per patient over the existing intervention alternatives. This approach follows the rationale that physicians are obliged to provide the best available medicine to their patients, but may prevent achieving equity in adoption of medical innovations. Buchanan et al. (27) use medical therapies as one of the best examples of injustice of innovation. They claim that justice in innovation is not restricted to the just distribution of existing beneficial innovations, for two reasons. First, as the case of essential medicines makes clear, the fact that important innovations are not occurring can be a concern of justice. If justice implies a human right to healthcare, this situation is unjust. Medicines that could save the lives of millions of people in these countries, at relatively low cost are not being developed. The conclusions of the PURE study (5), that mainstream adoption of low cost interventions could provide better outcomes for the target population, despite providing less than optimal therapy at the individual patient level, are in line with our objective and thesis.

### Model limitations

Our suggested approach may have several limitations. First, it has a major limitation when compared to the standard cost-effectiveness analysis as it compares therapies for a specific target population. CEA can assist in resource allocation among competing interventions for various disease areas and interventions, using a common outcome metric (e.g. QALY). However, the model could be extended to compare the outcomes of different target populations, by comparing the added QALYs achieved in each target population. Our approach has been used by the German healthcare system, which evaluates innovative interventions only in comparison to true alternatives for the same intended use population [26]. Our model uses only direct healthcare costs, and did not account for indirect costs, as is sometimes done in CEA. The rational was that the perspective taken here is of the payer who holds the budget, and the guidelines for budget impact analyses suggest modeling only direct costs [21]. It is possible, however, to extend the model to include indirect costs as well. For reasons of simplicity, we have not included in the model various parameters that may have a significant impact on costs and outcomes, such as adoption rates of the interventions, adherence, horizon of therapy and economic analyses and risk factors. It is possible to extend the model to include these parameters, and perform various sensitivity analyses on these parameters accordingly.

It may seem that our suggested model may under-estimate the value of an expensive intervention with long-term effects such as an effective vaccination, as a result of our focus on a fixed budget. However, assuming that the payer uses our proposed model with a relatively long term horizon to inform decisions, the model will demonstrate the benefits of herd immunity when fewer cases are arising each year.

Notwithstanding these limitations, our proposed framework may have important policy and industry implications. Since widespread diffusion of innovation is critical in order to make a significant impact on public health, payers must consider the effect of budget and price on market access to innovation.

## Conclusions

**Fig. 2:**
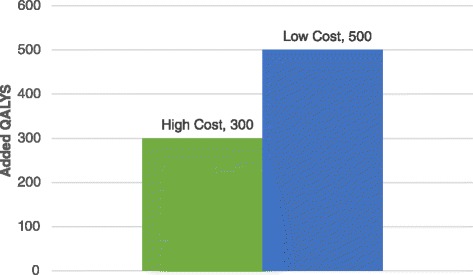
Comparison of added QALYs

Our proposed framework may have important policy and industry implications. The model provides a method for evaluating the health effects of treatment alternatives on an entire IUP, under a constrained budget. In the current cost-effectiveness paradigm, technologies are rated according to their ICER for a base case where an incremental cost over an incremental benefit is presented. This approach can ensure that the effectiveness of various technologies can be compared on the entire IUP. Since widespread diffusion of innovation is critical in order to make a significant impact on public health, payers must consider the effect of budget and price on market access to innovation. Although an intervention associated with higher costs and better outcomes may have an acceptable ICER, it could provide inferior outcomes under budget constraints, depending on the relative effectiveness and costs of the two interventions, as was suggested in the literature [27]. Fig. 2 illustrates the results in the demo scenario:

Moreover, higher costs of a drug may imply higher co-payments, which have a negative effect on adherence [28], and thus a negative impact on outcomes, as adherence has a significant impact on outcomes, as has been shown to be the case in secondary prevention post myocardial infarction [29]. The cost premium that can be justified for a more effective intervention is directly correlated to the effectiveness premium of that intervention over the lower cost/lower effectiveness intervention. In cases that a new technology does provide better outcomes for the intended use population, disinvestment in the old technology would be required.

The proposed policy could be applied to various technologies in various countries, in order to assist policy makers and payers to maximize the health outcomes of various intended use populations. The research should be carried out for each country separately, because of different patient populations, and different pricing schemes.
